# Development of a Predictive Model for Transfusion‐Related Acute Lung Injury Based on Neutrophil Extracellular Traps (NETs)

**DOI:** 10.1155/mi/7778073

**Published:** 2026-07-21

**Authors:** Qiong Wang, Zhenyang Li, Junliang Shao, Xinchen Qiang, Wen Gong, Lingling Sun, Huaying Yang, Zhen Li, Junfang Wang

**Affiliations:** ^1^ Department of Blood Transfusion, The Affiliated Wuxi People’s Hospital of Nanjing Medical University, Wuxi People’s Hospital, Wuxi Medical Center, Nanjing Medical University, Wuxi, Jiangsu, 214023, China, njmu.edu.cn; ^2^ Department of General Surgery, Huashan Hospital, Fudan University, Shanghai 200040, China, fudan.edu.cn; ^3^ Departments of Orthopedics, Wuxi People’s Hospital Affiliated to Nanjing Medical University, Wuxi Medical Center, Nanjing Medical University, Wuxi, Jiangsu, 214023, China, njmu.edu.cn

**Keywords:** acute lung injury, acute massive hemorrhage, neutrophil extracellular traps (NETs), transfusion

## Abstract

**Background:**

Patients receiving massive transfusion after acute hemorrhage are at risk for transfusion‐related acute lung injury (TRALI), a severe complication. Neutrophil extracellular traps (NETs) play a key role in acute lung injury. This study aimed to explore the link between NETs and TRALI and to develop a risk‐prediction model using machine learning for early detection and intervention.

**Methods:**

In this multicenter prospective study, 513 patients with acute massive hemorrhage who underwent transfusion therapy (March 2020–February 2025) were consecutively recruited. All biomarker assays, sampling time points, and the statistical analysis plan were prespecified and ethically approved before study initiation. Based on TRALI occurrence after transfusion, they were divided into an injured group (*n* = 42) and a uninjured group (*n* = 471). Clinical features and NET‐related markers were compared. LASSO regression was used for variable selection, followed by random forest for importance ranking. Multivariate logistic regression identified independent predictors, and a nomogram model was built and evaluated using ROC analysis, calibration, and decision curve analysis.

**Results:**

The injured group had significantly higher rates of smoking history, total infusion volume, perioperative transfusion volume, and transfusion frequency (all *p* < 0.05). Levels of citrullinated histone H3 (citH3), myeloperoxidase (MPO), neutrophil elastase (NE), interleukin‐6 (IL‐6), interleukin‐1*β* (IL‐1*β*), tumor necrosis factor‐*α* (TNF‐*α*), and interleukin‐8 (IL‐8) were also elevated (all *p* < 0.05). LASSO identified seven key variables, with citH3 and MPO showing high importance. Multivariate analysis confirmed citH3 (OR = 1.142), MPO (OR = 5.017), and NE (OR = 1.014) as independent predictors of TRALI (all *p* < 0.05). The combined model achieved an AUC of 0.85 (95% CI: 0.78–0.92), indicating strong predictive performance.

**Conclusion:**

TRALI risk in acute massive hemorrhage patients is associated with NET‐related markers, particularly citH3, MPO, and NE. A model integrating these indicators provides valuable early identification of TRALI, aiding clinical decision‐making.

## 1. Introduction

Acute massive hemorrhage represents a frequent and severe condition in clinical practice. It is often caused by trauma, surgery, or hemorrhagic disorders and is characterized by rapid and substantial blood loss within a short period, which results in a sharp reduction in blood volume and a cascade of severe consequences, including circulatory dysfunction, organ ischemia and hypoxia, and metabolic disturbances [1]. Without timely and effective intervention, patients can quickly progress to hypovolemic shock, with severe cases posing a life‐threatening risk and a reported mortality rate of 30%–50% [2]. Transfusion therapy is widely used in the management of acute massive bleeding to correct hemorrhagic shock, improve tissue perfusion, and prevent multiple‐organ injury. Previous reports have shown that transfusion can rapidly reverse circulatory collapse and correct anemia and coagulopathy, thereby markedly reducing shock‐related mortality [3]. Other studies indicate that administering fresh frozen plasma, platelets, or cryoprecipitate helps replenish coagulation factors, address coagulopathic abnormalities, and control ongoing bleeding [4]. However, clinical observations reveal that some patients still develop transfusion‐related acute lung injury (TRALI) during transfusion, with an incidence of 3.8%−7.2% [5]. Once TRALI occurs, both the length of the ICU stay and the 30‐day mortality increase significantly [6]. Therefore, identifying risk factors for TRALI following massive transfusion in acute hemorrhage patients and establishing a predictive model are essential for guiding clinical decision‐making and optimizing subsequent treatment strategies.

Following the onset of TRALI in patients with acute massive hemorrhage, inflammatory markers in the body rise markedly, and these elevations are closely linked to disease severity and prognosis [7]. Published research suggests that, when compared to healthy individuals in the control group, patients diagnosed with TRALI seem to have significantly higher plasma concentrations of specific cytokines. These cytokines include interleukin‐6 (IL‐6), interleukin‐1*β* (IL‐1*β*), and tumor necrosis factor‐*α* (TNF‐*α*) [8]. Other research has shown that interleukin‐8 (IL‐8) is also highly expressed in TRALI and serves as an important indicator for assessing disease severity [9]. Thus, traditional clinical assessment has often relied on IL‐6, IL‐1*β*, TNF‐*α*, and IL‐8 to evaluate the severity of TRALI. However, increasing evidence indicates that these conventional inflammatory markers lack specificity and are easily influenced by non‐TRALI factors, limiting their predictive value for TRALI [10]. In this context, neutrophil extracellular traps (NETs)‐related markers have gained attention due to their reported association with TRALI [11]. NETs are commonly regarded as net‐like formations that are discharged by neutrophils when they encounter various types of stimulation. In the case of TRALI, it has been noted that related markers, such as citrullinated histone H3 (citH3), myeloperoxidase (MPO), and neutrophil elastase (NE), show increased levels. Measuring these markers can be useful for distinguishing TRALI from other kinds of acute lung injury. citH3 is produced when histone H3 undergoes citrullination catalyzed by peptidylarginine deiminase 4, altering its charge distribution, weakening DNA binding, and loosening the chromatin structure—an essential step in NET formation. MPO, a neutrophil‐derived enzyme with bactericidal activity, also participates in cardiovascular pathology; abnormal levels indicate the early accumulation of harmful substances before clinical manifestations of heart disease. NE, a serine protease stored in neutrophil granules, exhibits broad substrate specificity and can degrade multiple extracellular matrix components and cell‐surface receptors [12, 13]. Although NET‐related markers show clear associations with TRALI, studies focusing on their ability to predict TRALI during transfusion in acute massive hemorrhage remain limited. The current research was carried out to explore the possible association between NETs and TRALI in patients who underwent transfusion treatment. Additionally, an attempt was made to build a risk prediction model for TRALI. This model was created by utilizing a random forest algorithm with the intention of attaining a strong predictive ability. The goal is to enable early identification and timely intervention for TRALI, thereby supporting more effective clinical decision‐making.

## 2. Methods

### 2.1. Study Subjects

A prospective study was conducted involving 513 patients with acute massive hemorrhage who received transfusion therapy at Wuxi People’s Hospital and Huashan Hospital. The current analysis incorporated patients from two medical establishments, Fudan University and Wuxi People’s Hospital affiliated with Nanjing Medical University, spanning from March 2020 to February 2025. Patient enrollment and data acquisition were conducted prospectively: each patient was enrolled at the time of admission, with synchronous collection of clinical data and transfusion‐related parameters, prospective measurement of NETs and inflammatory markers, and follow‐up monitoring for TRALI after transfusion (all biomarker assays were performed before transfusion to ensure that the timeline conformed to predictive‐model logic). In this prospective investigation, the patient cohort was divided into two separate clinical categories according to the posttransfusion clinical outcome of TRALI occurrence. Specifically, 42 patients who experienced TRALI were allocated to the injured group, whereas 471 patients without TRALI were designated as the uninjured group. The study protocol underwent a review process and was eventually sanctioned by the institutional ethics committees at the participating facilities. The research was conducted in line with the ethical guidelines set forth in the Declaration of Helsinki.

### 2.2. Diagnostic Criteria

The study necessitated that all the patients who were enrolled had to satisfy the diagnostic benchmarks for acute large‐scale bleeding. These benchmarks were established by the expert consensus on acute massive hemorrhage [14]: (1) blood loss ≥1 L within 1 h; (2) heart rate (HR) >110 beats/min, systolic blood pressure (SBP) <90 mmHg, mean arterial pressure (MAP) <65 mmHg, or urine output <0.5 mL/kg·h; and (3) hemoglobin reduction ≥2 g/dL, prothrombin time (PT) >1.5 times the normal value, or platelet count <50 × 10^9^/L.

### 2.3. Inclusion and Exclusion Criteria

#### 2.3.1. Inclusion Criteria

(1) The main inclusion criterion stipulated that every patient had to fulfill the diagnostic standards for acute severe bleeding. These standards were the ones detailed in the previously mentioned expert consensus paper; (2) fulfilling the indications for transfusion and receiving transfusion therapy; (3) absence of pulmonary symptoms, such as cough, chest tightness, or dyspnea, within 72 h before transfusion, with a normal chest X‐ray; and (4) another prerequisite was the accessibility of comprehensive medical histories and subsequent follow‐up details for every patient.

#### 2.3.2. Exclusion Criteria

(1) History of pulmonary infection within 14 days before transfusion; (2) presence of hematologic malignancy or congenital coagulation disorders; (3) existing pulmonary hypertension, sepsis, or pulmonary contusion; and (4) allergy to blood product components or interruption of transfusion due to severe arrhythmia, heart failure, or other serious complications.

### 2.4. Data Collection

Patient medical records were collected through the hospital’s information management system and compiled into an Excel spreadsheet. The data set that had been gathered included patient‐related variables. These variables covered fundamental demographic details, for instance, gender, age, and body mass index (BMI). Moreover, clinical and behavioral elements were documented. These elements incorporated smoking background, alcohol consumption, prior cases of diabetes mellitus, past history of hypertension, mechanical breathing support, the reason for bleeding, pretransfusion shock status, the overall fluid quantity, the volume of blood transfused during the perioperative period, the number of blood transfusions carried out, and the kinds of blood products given.

### 2.5. Transfusion Therapy and Fluid Management

Peripheral venous access was routinely established in all patients. A solution of sodium lactate Ringer’s (Chenxin Pharmaceutical Co., Ltd., 500 mL, National Drug Approval Number H20057107) and 6% low‐molecular‐weight dextran colloid (Livzon Group Liming Pharmaceutical Factory, 250 mL, National Drug Approval Number H44025313) was mixed in a 2 : 1 ratio and administered at 10 ml·kg^−1^·h^−1^. Throughout fluid resuscitation, the blood pressure was maintained within the normal range. Concentrated red blood cells and plasma were transfused according to the patient’s blood loss and arterial blood gas results, while also considering cardiac and pulmonary function, metabolic status, and the degree of organ ischemia.

### 2.6. TRALI

All patients received transfusion therapy and were grouped according to the occurrence of TRALI: those who developed TRALI were assigned to the injured group, while patients without TRALI were placed in the uninjured group. TRALI was diagnosed based on the Revised International Expert Consensus on TRALI: (1) onset of symptoms during transfusion or within 6 h after transfusion; (2) bilateral pulmonary infiltrates on chest imaging without evidence of pulmonary vascular overload; (3) oxygenation index ≤40 kPa; and (4) absence of elevated left atrial pressure, with left atrial pressure <18 mmHg [15].

### 2.7. Measurement of NET‐Related Markers

In accordance with the prospectively prespecified detection plan in the study protocol, all blood samples were collected after admission and before initiation of transfusion, and NET‐related markers were measured immediately. Detection procedure: fasting (time since last meal ≥4 h) venous blood (5 mL) was collected and allowed to stand at room temperature for 30 min. Samples were then centrifuged at 4000 r/min for 10 min with a centrifuge radius of 8 cm to separate the serum. An assessment of NETosis indicators, such as citH3, MPO, and NE, was carried out. This evaluation employed a fluorescence immunoassay approach. The measurements were executed on an analyzer, specifically the CliPAH301 model.

### 2.8. Measurement of Inflammatory Markers

In accordance with the prospectively prespecified detection plan in the study protocol, all blood samples were collected after admission and before initiation of transfusion, and inflammatory markers were measured immediately. Detection procedure: a 5‐mL fasting (time since last meal ≥4 h) venous blood sample was taken from each participant. After collection, the blood samples were allowed to sit at room temperature for half an hour. Next, the samples underwent a centrifugation process. Usually, this centrifugation was done at 1200 rotations per minute for 10 min using a centrifuge with an 8‐cm radius to isolate the serum part. After that, the concentrations of specific cytokines in the serum were measured. These cytokines included IL‐6, IL‐1*β*, TNF‐*α*, and IL‐8. The quantification was accomplished via an ELISA. The assay was carried out following the guidelines provided by the manufacturer of the commercial kit, which was obtained from Sigma in the United States.

### 2.9. Statistical Analysis

GraphPad Prism 9 (for between‐group comparisons of quantitative and qualitative data) and R software 4.2.1 (for LASSO regression, random forest, XGBoost, nomogram construction, ROC analysis, calibration curves, and decision curve analysis; R packages “glmnet,” “randomForest,” “xgboost,” “rms,” “pROC,” and “rmda”) were utilized to conduct statistical analysis. The Shapiro–Wilk test was applied to assess the normality of the distribution of continuous variables. Subsequently, Levene’s test was used to verify the homogeneity of variance. For variables that were determined to have a normal distribution and equal variance, the data are presented as the mean ± standard deviation (SD). When comparing two independent groups, an independent‐samples *t*‐test was usually used. On the other hand, a paired‐samples *t*‐test was typically employed for comparisons within a single group. Data that did not follow a normal distribution are expressed as the median along with the interquartile range [M(P_25_, P_75_)]. The Mann–Whitney *U* test was used to analyze these nonnormally distributed variables. Categorical variables are shown as counts and percentages [n (%)]. The chi‐square (*χ* ^2^) test was used to perform group comparisons for these categorical variables. Variables showing statistically significant differences were included in LASSO and multivariate logistic regression analyses to identify factors influencing the development of TRALI in patients receiving massive transfusion for acute hemorrhage. In this analysis, a significance threshold of *α* = 0.05 was utilized for all statistical tests carried out. As a result, a *p*‐value discovered to be lower than 0.05 was typically regarded as an indication of a statistically significant outcome.

## 3. Results

### 3.1. The Clinical Features Were Contrasted Between the Group With Injuries and the Group Without Injuries

When examining the clinical features of the two groups, it was noted that 69.05% of patients in the injured group reported a smoking history. This percentage was significantly higher than that of the uninjured group, where only 36.73% of patients had a similar history, and the *p*‐value was less than 0.05. In terms of fluid handling, the total amount of fluid infused in the injured group was computed to be 3,125.58 mL. This volume exceeded that of the uninjured group, which had a recorded infusion volume of 2,763.42 mL, and this difference was statistically significant. Moreover, there was a disparity in the perioperative blood transfusion volume between the two groups. The injured group received 2,307.16 mL of blood, while the uninjured group received ~2055.89 mL. Additionally, the data showed that 69.05% of patients in the injured group had three or more blood transfusions. This contrasted sharply with the uninjured group, where only 33.97% of patients had three or more transfusions, as shown in Table 1.

**Table 1:** A comparison of the clinical features was carried out between the group with injuries and the group without injuries.

Clinical characteristics	Injured group (*n* = 42)	Uninjured group (*n* = 471)	*χ* ^2^/*t*	*P*
Sex				
Male	25	263	0.213	0.645
Female	17	208		
Age ($\overset{―}{\chi }$ ± s, years)	54.69 ± 9.37	55.10 ± 10.14	0.253	0.801
BMI ($\bar{\chi }$ ± s, kg/m^2^)	23.72 ± 1.25	23.85 ± 1.60	0.513	0.608
Smoking history				
Yes	29	173	16.871	<0.001
No	13	298		
Drinking history				
Yes	22	204	1.287	0.257
No	20	267		
Diabetes history				
Yes	14	193	0.936	0.333
No	28	278		
Hypertension history				
Yes	11	165	1.337	0.247
No	31	306		
Mechanical ventilation				
Yes	16	174	0.022	0.882
No	26	297		
Cause of bleeding				
Trauma	13	138	1.904	0.386
Surgery	17	151		
Hemorrhagic disease	12	182		
Shock before transfusion				
Yes	9	86	0.257	0.612
No	33	385		
Total infusion volume ($\bar{\chi }$ ± s, mL)	2811.58 ± 137.21	2763.42 ± 120.98	2.444	0.015
Perioperative transfusion volume ($\bar{\chi }$ ± s, mL)	2090.16 ± 118.25	2055.89 ± 101.44	2.068	0.039
Number of transfusions				
<3 times	13	311	20.391	<0.001
≥3 times	29	160		
Blood product type				
Cryoprecipitate	16	175	0.237	0.888
Fresh frozen plasma	19	203		
Apheresis platelets	7	93		

### 3.2. Contrast of NET‐Associated Indicators Between the Injured Group and the Noninjured Group

**Figure 1:**
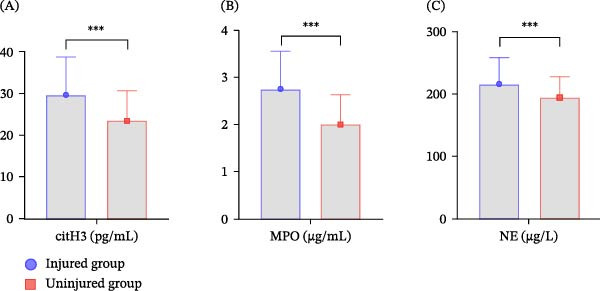
A comparison was made of NET‐associated indicators between the injured group and the noninjured group. Specifically, this involved: (A) a comparison of citrullinated histone H3 (citH3) levels between the two groups; (B) an assessment of myeloperoxidase (MPO) differences between the two groups; and (C) an examination of neutrophil elastase (NE) disparities between the two groups.  ^∗∗∗^ *p* < 0.001.

NETs serve as biomarkers, reflecting the severity of a patient’s condition. To enable a comparison between the injured and uninjured groups, the quantification of markers associated with NETs, namely, citH3, MPO, and NE, was carried out. In the injured group, the measured level of citH3 was 29.87 pg/mL. Remarkably, this value was significantly greater than the 23.56 pg/mL level detected in the uninjured group, as indicated by a calculated *p*‐value less than 0.05. MPO levels were 2.43 μg/mL in the injured group versus 1.98 μg/mL in the uninjured group, also showing a significant difference (*p* < 0.05). NE levels were 216.52 and 190.43 μg/L in the injured and uninjured groups, respectively, with the injured group being higher (*p* < 0.05, Figure 1).

### 3.3. Contrast of Inflammatory Markers Between the Injured Group and the Noninjured Group

**Figure 2:**
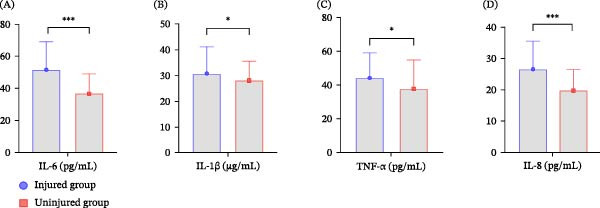
Contrast of inflammatory markers between the injured group and the noninjured group (A) contrast of interleukin‐6 between the two groups; (B) contrast of interleukin‐1 beta between the two groups; (C) contrast of tumor necrosis factor‐alpha between the two groups; and (D) contrast of interleukin‐8 between the two groups.  ^∗^ *p* < 0.05,  ^∗∗∗^ *p* < 0.001.

Inflammatory markers are important indicators for evaluating the intensity of a patient’s inflammatory response. When evaluating the inflammatory state in two patient groups, the serum concentrations of IL‐6, IL‐1*β*, TNF‐*α*, and IL‐8 were determined. The data showed that in the injury cohort, the IL‐6 level reached 51.47 pg/mL, which was markedly higher than 36.89 pg/mL measured in the noninjury cohort (*p* < 0.05). Likewise, the IL‐1*β* level in the injured group was 30.85 pg/mL, while in the uninjured group, it was 22.37 pg/mL. This disparity was also statistically significant. Regarding TNF‐*α*, the measured concentrations were 44.36 pg/mL in the injured group and 40.17 pg/mL in the uninjured group, indicating higher levels in the injured group. Moreover, the IL‐8 level in the injured group was 26.52 pg/mL, significantly exceeding the 19.68 pg/mL found in the uninjured group (*p* < 0.05). These comparison results are depicted in Figure 2.

### 3.4. Preliminary Variable Selection Using LASSO Regression

**Figure 3:**
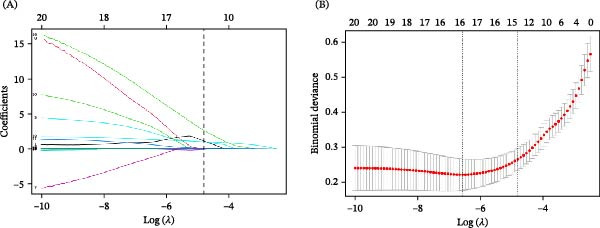
Selection of clinical features based on the LASSO regression model (A) LASSO regression coefficient path; (B) 10‐fold cross‐validation MSE plot; *x*‐axis: log(*λ*); *y*‐axis: variable coefficient/cross‐validation error.

During the analysis, the occurrence of TRALI subsequent to massive blood transfusion in patients experiencing acute hemorrhage was commonly utilized as the dependent variable. To select the variables, a LASSO regression model was implemented. All elements that had shown statistical significance in previous univariate analyses were incorporated into this model. Usually, a 10‐fold cross‐validation method was utilized to ascertain the optimal value for the tuning parameter *λ*. It was noted that as the penalty parameter *λ* grew, the coefficients related to the independent variables were gradually reduced to approach zero. The *λ* value corresponding to the minimum cross‐validation error, which was 0.002, was generally regarded as the optimal parameter. Through this process, seven variables were identified as seemingly crucial influencing factors. These factors were the total volume of infusion, the frequency of transfusions, citH3, MPO, NE, IL‐1*β*, and TNF‐*α*, as depicted in Figure 3.

### 3.5. Variable Importance Ranking Based on the Random Forest Algorithm

**Figure 4:**
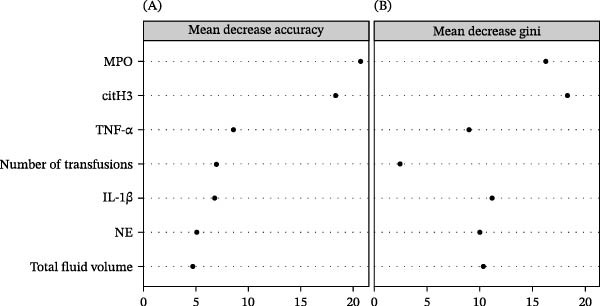
Bar plots of the relative importance of variables in the models (A) XGBoost. (B) Random forest. *x*‐axis: importance score; *y*‐axis: variable name.

The importance of key variables was ranked using the XGBoost model and the random forest algorithm. In the XGBoost model, the relative importance of variables was ranked as MPO > citH3 > TNF‐*α* > number of transfusions > IL‐1*β* > NE > total infusion volume (Figure 2A). In the random forest model, the ranking was citH3 > MPO > IL‐1*β* > NE > total infusion volume > TNF‐*α* > number of transfusions (Figure 4). Notably, citH3 and MPO showed particularly high importance, indicating their central predictive value in identifying the TRALI risk.

### 3.6. Multivariate *Logistic* Regression Model Construction

Subsequently, the seven factors chosen via the LASSO regression model were integrated into a multivariate logistic regression analysis. To identify the independent risk factors for TRALI after massive transfusion in patients experiencing acute hemorrhage, a step‐by‐step regression method was then applied. The results of this analysis indicated that citH3 (odds ratio = 1.142, 95% confidence interval: 1.082–1.206), MPO (odds ratio = 5.017, 95% confidence interval: 2.668–9.435), and NE (odds ratio = 1.014, 95% confidence interval: 1.003–1.026) seemed to be independent predictors of TRALI in this clinical setting. The *p*‐values for these factors were less than 0.05, as specifically shown in Table 2.

**Table 2:** Construction of the multivariate logistic regression model.

Factor	Regression Coefficient	Standard Error	*z*‐Value	*p*‐Value	OR	95% CI for OR
Total infusion volume	0.003	0.002	1.818	0.069	1.003	1.000~1.007
Number of transfusions	0.005	0.002	1.947	0.052	1.005	1.000~1.010
citH3	0.133	0.028	4.786	<0.001	1.142	1.082~1.206
MPO	1.613	0.322	5.005	<0.001	5.017	2.668~9.435
NE	0.014	0.006	2.439	0.015	1.014	1.003~1.026
IL‐1β	0.047	0.026	1.791	0.073	1.048	0.996~1.104
TNF‐α	0.023	0.013	1.754	0.079	1.023	0.997~1.049

### 3.7. Nomogram Model Visualization

**Figure 5:**
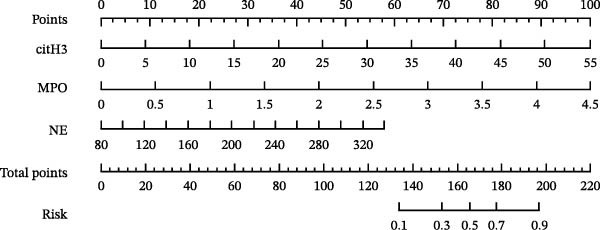
Following the results derived from the multivariate logistic regression analysis, a nomogram prediction model was then constructed to assess the risk of transfusion‐related acute lung injury (TRALI) in patients who had experienced acute massive hemorrhage after undergoing massive transfusion.

Following the results procured from the multivariate logistic regression analysis, a nomogram prediction model was then constructed: Logit(P) = −1.872 + 0.133 × citH3 + 1.613 × MPO + 0.014 × NE. Using these three selected predictors, a visual scoring system was developed. Each risk factor is represented by an independent axis, with the length of the scale reflecting the relative contribution of that factor to the outcome. The points corresponding to each variable are summed to obtain a total score, which is then mapped onto a probability scale to generate an individualized risk estimate. This system allows for a quantitative assessment of TRALI risk in patients receiving massive transfusion for acute hemorrhage (Figure 5).

### 3.8. ROC Curve, Calibration Plot, and Decision Analysis Curve for the Validation of the Model

**Figure 6:**
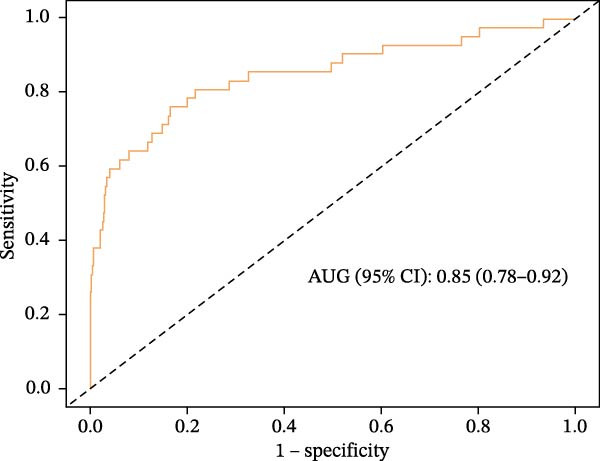
The area beneath the receiver operating characteristic (ROC) curve for the nomogram model.

**Figure 7:**
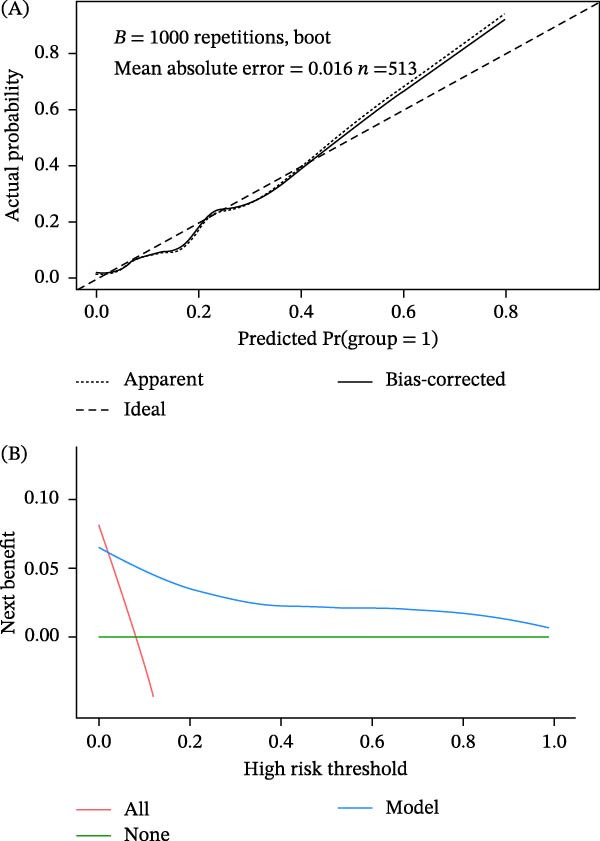
Calibration plot and decision plot of the nomograph model (A) the *x*‐axis shows the TRALI risk probability predicted by the nomogram and the *y*‐axis the actual observed proportion of TRALI; the diagonal represents the ideal prediction line and the solid line the bias‐corrected model curve; points indicate the observed event rate of each risk‐stratified subgroup, and the closer they lie to the diagonal, the greater the predictive accuracy. (B) the *x*‐axis represents the high‐risk threshold and the *y*‐axis the net benefit; “None” indicates no intervention, “All” indicates intervention in all patients, and “Model” indicates intervention guided by the present nomogram; the risk score refers to the individualized TRALI risk calculated from citH3, MPO, and NE.

When examining the receiver operating characteristic curve, it was noted that the combined prediction of TRALI using the biomarkers citH3, MPO, and NE resulted in an area under the curve of 0.85. The 95% confidence interval for this area ranged from 0.78 to 0.92, with a sensitivity of 0.76 (0.63–0.89) and a specificity of 0.83 (0.80–0.87); precision was 0.90, recall was 0.91, and the F1 score was 0.89. As illustrated in Figure 6, this outcome implies that the developed nomogram model has fairly good discriminatory capacity. To validate the model, a bootstrap resampling technique with 1,000 repetitions was employed. The Nagelkerke *R* ^2^ value was 0.325, indicating that the model has a strong explanatory power for the dependent variable and suggesting that this column chart model shows good calibration. As shown in Figure 7A, the *x*‐axis represents the TRALI risk predicted by the nomogram, and the *y*‐axis the actual observed proportion of TRALI; each plotted point corresponds to the observed event rate of a risk‐stratified subgroup, and the closer the points lie to the diagonal, the better the agreement between the predicted and observed risk. Moreover, decision curve analysis was carried out. As shown in Figure 7B, the results of this analysis revealed that, across a variety of threshold probabilities, the nomogram offers a greater net clinical benefit compared to extreme reference strategies; here, the risk score, derived from the nomogram, represents the individualized predicted probability of TRALI, intended to guide the identification of high‐risk patients and early intervention. This discovery suggests that the factors incorporated into the final model possess a significant predictive value. To further evaluate model robustness, a supplementary multivariate model incorporating key confounders, namely, smoking history, total infusion volume, perioperative transfusion volume, transfusion frequency, and core NET markers, was constructed using a forced‐entry multivariate logistic regression regardless of univariate significance in order to control for confounding. The supplementary model yielded an AUC of 0.88, and the DeLong test comparing the two models’ AUCs showed a *p* > 0.05, indicating that the difference in predictive performance between the two models was not statistically significant.

## 4. Discussion

According to data, transfusion provides effective treatment for patients with acute massive hemorrhage; however, 5–8 out of every 100 patients receiving massive transfusion may develop TRALI [16]. TRALI is a serious transfusion‐related complication and a significant contributor to transfusion‐associated mortality. It typically occurs during transfusion or within 6 h afterward, presenting clinically as acute noncardiogenic pulmonary edema. The condition is characterized by rapid progression and complex clinical manifestations, posing a serious threat to patient survival [17].

Studies have shown that, compared with nonsmokers, patients with a history of smoking may face a higher perioperative risk of TRALI‐related mortality [18]. In the present study, the injured group exhibited a significantly greater incidence of smoking history compared with the uninjured group, suggesting that smoking may promote TRALI development. Prolonged smoking can cause structural and functional changes in the lungs, impair pulmonary function, increase systemic inflammation, and disrupt immune function. This reduces the lungs’ ability to respond to external stimuli and recover, making them more susceptible to injury. In addition, harmful substances in cigarette smoke, such as nicotine and carbon monoxide, can affect blood oxygenation, further increasing the risk of TRALI [19]. The results of the analysis showed that, when contrasted with the noninjury cohort, the injured group had larger overall infusion amounts, higher perioperative blood transfusion volumes, and a greater quantity of transfusion occurrences. These findings might appear to imply that the delivery of substantial volumes of fluid and a more frequent rate of transfusions could be linked to the onset of TRALI. High total infusion volumes can elevate colloid osmotic pressure, promote capillary leakage, exacerbate pulmonary edema, and accelerate disease progression, all of which may facilitate TRALI onset. Large fluid volumes may also dilute coagulation factors, increasing the bleeding risk and further predisposing patients to TRALI [20]. With increasing perioperative transfusion volumes, the total load of TRALI‐inducing substances rises, directly stimulating neutrophils, promoting massive NET release, and obstructing the pulmonary microvasculature. Massive transfusion can also cause dilutional coagulopathy, activating the thrombin–inflammatory factor pathway and aggravating damage to the pulmonary epithelial/endothelial barrier [21, 22]. Additionally, repeated transfusions lead to recurrent immune stimulation and cumulative risk exposure, activating inflammatory memory and directly inducing a TRALI‐susceptible state, thereby significantly increasing the TRALI incidence [23]. During this investigation, it was noted that there seemed to be significant disparities between the injured and noninjured patient groups when considering the patients’ smoking histories, total infusion volume, perioperative transfusion volume, and number of transfusions. These factors were not included in the subsequent risk factor model, possibly due to data limitations or time‐dependent bias.

Prior clinical studies have indicated that frequently measured markers of inflammation, including IL‐6, IL‐1*β*, TNF‐*α*, and IL‐8, typically seem to be increased in instances of TRALI. These findings generally imply a possible association between these cytokines and the clinical state [24]. During the current analysis, it was noted that the measured levels of IL‐6, IL‐1*β*, TNF‐*α*, and IL‐8 seemed to be significantly higher in the injured group as compared to the noninjured group, indicating an upward trend of these markers in TRALI patients. These cytokines contribute to TRALI pathogenesis mainly through the regulation of inflammatory cascades, neutrophil activation, and disruption of the pulmonary barrier. Further analysis revealed that IL‐6 exacerbates alveolar–capillary barrier damage by inducing acute‐phase protein synthesis and neutrophil infiltration, and its expression closely correlates with the oxygenation index in TRALI patients. IL‐1*β* activates the coagulation system and complement pathways, enhances the inflammatory response, and promotes neutrophil degranulation and NET release, directly injuring pulmonary vascular endothelium and increasing TRALI risk. TNF‐*α* damages pulmonary endothelial cells and increases capillary permeability, with levels associated with the severity of pulmonary edema. IL‐8, a key neutrophil chemoattractant, is influenced by the degree of pulmonary neutrophil infiltration and TRALI severity [25, 26]. Thus, IL‐6, IL‐1*β*, TNF‐*α*, and IL‐8 are closely related to TRALI; however, their predictive utility for TRALI remains limited. Of note, in assessing TRALI severity, the anti‐inflammatory cytokine interleukin‐10 (IL‐10) also has an important role as a reference value. Recent clinical evidence has demonstrated that IL‐10 levels are significantly reduced in TRALI patients, suggesting that an inadequate anti‐inflammatory response aggravates pulmonary tissue injury and that IL‐10 may serve as an important indicator for evaluating TRALI severity [27].

In recent years, a growing body of animal evidence has identified a critical pathogenic role of NETs in TRALI, providing a biological basis for the present findings [28]. In a murine TRALI model [29], transfusion of blood products containing anti‐HLA/anti‐MHC class I antibodies rapidly activated the complement C3a/C5a pathway, thereby inducing neutrophil activation and massive NET release; complement activation acts as a key upstream signal triggering NET formation, and blockade of the C5a receptor markedly reduces pulmonary NET deposition and attenuates lung injury. Accordingly, NETs, via the release of citH3, MPO, and NE, can directly disrupt the pulmonary microvascular endothelial barrier, increasing endothelial permeability and promoting pulmonary edema; concurrently, NETs facilitate platelet adhesion and aggregation, leading to pulmonary microvascular microthrombosis and aggravating perfusion disturbance and hypoxemia.

Furthermore, NETs have been increasingly recognized for their strong link to the onset and progression of diseases and their critical involvement in TRALI pathogenesis [27]. Consistent with the previously mentioned findings, the present analysis seemed to suggest that the measured values of NETs biomarkers, specifically citH3, MPO, and NE, were higher in the injured group compared to the noninjured group. According to the analytical techniques utilized, this disparity in biomarker concentrations was found to be statistically significant. Furthermore, citH3, MPO, and NE emerged as independent risk factors for TRALI following massive transfusion in patients with acute hemorrhage, confirming their association with TRALI development. Analysis indicates that citH3, a key component of NETs, is typically released from activated neutrophils. While NETs help capture pathogens and contribute to host defense, excessive NET release can damage pulmonary endothelium, increase capillary permeability, disrupt the alveolar–capillary barrier, and allow inflammatory mediators to extravasate, thereby exacerbating lung injury and increasing TRALI risk [29]. MPO is widely regarded as a principal enzyme present in neutrophil granules. It is believed that this enzyme participates in the production of reactive oxidant substances, such as hypochlorous acid, and it is linked to powerful antimicrobial properties. However, excessive MPO activity can trigger oxidative stress and inflammation, directly injuring lung tissue and promoting TRALI. NE, a protease, effectively degrades extracellular matrix components, contributing to pulmonary endothelial barrier disruption. NE also activates protease‐activated receptors, further amplifying inflammation, damaging lung tissue, and triggering inflammatory cascades, thereby participating in TRALI pathogenesis [28]. Based on these findings, a risk prediction model was developed. The combined prediction using citH3, MPO, and NE demonstrated a significant predictive value for TRALI. The nomogram translates the complex statistical model into a visual tool, providing individualized risk probabilities to aid clinical decision‐making. Consequently, the outcomes of the ROC curve analysis seemed to show a decent capacity to distinguish the risk of TRALI by jointly utilizing the biomarkers citH3, MPO, and NE. This discovery might suggest that a model founded on these markers could aid in the early detection of patients at high risk. In turn, this could facilitate more prompt guidance of clinical interventions [29].

This study has several limitations. First, it included only 513 acute massive hemorrhage patients treated at our institution, although it was part of a multicenter collaborative effort. Differences in equipment, staff experience, and clinical practices across centers may have led to variability in data quality, affecting the reliability of the results. Second, variations in patient baseline characteristics, disease severity, and treatment practices across regions may act as confounding factors, reducing the interpretability of the findings. Third, NET‐related biomarkers were measured only once, prior to transfusion, without continuous dynamic monitoring during or after transfusion, so the temporal trajectory of these markers between transfusion and TRALI onset cannot be characterized. Fourth, although this study identified an association between citH3, MPO, NE, and TRALI, causal relationships were not verified through interventional experiments. Moreover, smoking history and transfusion‐related variables were imbalanced between groups; although they were not retained by LASSO in the final model, their potential confounding effects were not formally excluded by sensitivity analysis, and the independent predictive value of NET markers should therefore be validated in larger prospective cohorts. In addition, with only 42 TRALI cases versus 471 non‐TRALI cases, the marked class imbalance may have biased the model toward the majority class, potentially leading to overestimation of the AUC and reduced recognition of the minority class; techniques such as SMOTE oversampling, weighted loss functions, or stratified cross‐validation were not used to address this imbalance, nor were balanced accuracy and precision–recall curves employed for model evaluation. Furthermore, only internal validation was performed: external‐cohort validation and temporal split validation were not carried out, and the model’s performance may degrade when applied to independent datasets. Finally, although LASSO regression, random forest, XGBoost, and multivariate logistic regression were employed to develop the predictive model, the modeling workflow is not fully transparent: it was not explicitly stated whether feature selection and model training were performed on the same dataset; the hyperparameter optimization procedure was not described in detail; the use of random forest and XGBoost for variable importance ranking rather than as the final predictive model was not clearly distinguished; and a model‐development flowchart and the explicit partitioning of training, validation, and test sets were not provided. Therefore, the study does not strictly conform to the TRIPOD reporting guideline, which may, to some extent, affect the methodological reproducibility and rigor of the work. To overcome these limitations, subsequent studies could adopt multicenter designs with larger sample sizes and a balanced case distribution to improve statistical power; incorporate longitudinal, multiple‐time‐point biomarker measurements to better characterize the temporal dynamics of NETs in relation to TRALI development; and perform sensitivity analyses in which all clinically relevant variables (smoking history, total infusion volume, transfusion frequency, perioperative transfusion volume, etc.) are forced into the model to further validate the independent predictive value of NET markers. In addition, animal experiments or in vitro models could be used to block NET formation or inhibit MPO/NE activity, thereby verifying causal relationships; meanwhile, external‐cohort validation or temporal split‐validation strategies should be introduced to refine the validation framework, and the TRIPOD reporting guideline should be strictly followed by supplementing dataset partitioning, hyperparameter tuning, clearly stated algorithm roles, and a model‐development flowchart so as to enhance transparency, reproducibility, and generalizability and thereby provide a stronger basis for the subsequent optimization of clinical treatment.

## 5. Conclusion

In conclusion, some patients with acute massive hemorrhage may develop TRALI during transfusion therapy. This study found that NET‐related markers—citH3, MPO, and NE—are key factors influencing TRALI occurrence. Drawing on the data amassed from this analysis, it is deemed viable to develop a risk prediction model for TRALI within the scope of transfusion therapy. This model would be instrumental in promptly pinpointing patients facing a greater risk. Once these patients are identified, it becomes possible to promptly introduce more tailored clinical interventions. These interventions, in turn, have the potential to enhance patient prognoses.

## Author Contributions

**Qiong Wang**: conceptualization, methodology, investigation, data curation, formal analysis, writing – original draft, writing – review and editing. **Zhenyang Li**: investigation, resources, data curation, validation. **Junliang Shao**: methodology, software, formal analysis, visualization. **Xinchen Qiang**: investigation, resources, project administration. **Wen Gong**: investigation, validation, data curation. **Lingling Sun**: investigation, methodology, validation. **Huaying Yang**: investigation, resources. **Zhen Li**: resources, supervision. **Junfang Wang**: conceptualization, methodology, resources, writing – review and editing, supervision, project administration, funding acquisition.

## Funding

This research was funded by the Wuxi City’s “Double Hundred” Young and Middle aged Medical and Health Top Talents (Grant BJ2023004) and the Jiangsu Province Preventive Medicine General Project (Grant Ym2023008).

## Disclosure

All aspects of the work, including study conception, data analysis, manuscript writing, and critical revision, were conducted solely by the authors.

## Ethics Statement

This study was reviewed and approved by the Ethics Committee of Wuxi People’s Hospital Afffliated to Nanjing Medical University (KY25230). Written informed consent was obtained from all participants, and all procedures followed the principles of the Declaration of Helsinki.

## Conflicts of Interest

The authors declare no conflicts of interest.

## Data Availability

The data that support the findings of this study are available upon request from the corresponding author. The data are not publicly available due to privacy or ethical restrictions.
